# Osteosarcoma immunometabolism: emerging mechanisms and clinical implications

**DOI:** 10.3389/fimmu.2025.1689790

**Published:** 2025-10-23

**Authors:** Bowen Tan, Jingyuan Ning

**Affiliations:** ^1^ Fifth Department of Orthopaedics, The Third Affiliated Hospital of Qiqihar Medical College, Qiqihar, China; ^2^ State Key Laboratory of Common Mechanism Research for Major Diseases & Department of Medical Genetics, Institute of Basic Medical Sciences & School of Basic Medicine, Chinese Academy of Medical Sciences and Peking Union Medical College, Beijing, China

**Keywords:** osteosarcoma, tumor microenvironment, immunometabolism, metabolic reprogramming, immunotherapy

## Abstract

Osteosarcoma (OS) is the most common primary malignant bone tumor, predominantly affecting adolescents and young adults. Despite decades of research, survival rates for metastatic or recurrent disease remain dismal, underscoring the urgent need for therapeutic innovation. This malignancy frequently exhibits refractory responses to immunotherapy, a limitation increasingly attributed to dysregulated immunometabolic crosstalk. Growing evidence supports cellular metabolism as a master regulator of both neoplastic progression and immune cell functionality. To meet heightened biosynthetic demands, OS cells undergo metabolic reprogramming, adopting distinct programs divergent from normal counterparts. These changes reshape the tumor microenvironment (TME) into an immunosuppressive milieu, restricting immune cell infiltration and effector activity. Consequently, targeting these immunometabolic pathways offers a promising strategy to overcome therapeutic resistance. Here, we critically analyze the current understanding of OS immunometabolism, systematically delineating OS-specific evidence from extrapolated concepts. We dissect the key metabolic barriers to successful immunotherapy and propose a forward-looking roadmap to guide the development of more effective, biomarker-driven therapeutic strategies.

## 1 Introduction

Osteosarcoma (OS) is a prototypical primary malignant bone tumor arising from aberrant differentiation of mesenchymal stem cells, characterized by the production of malignant osteoid (1). It most frequently occurs in the metaphyseal regions of long bones—particularly the distal femur, proximal tibia, and proximal humerus—and primarily affects adolescents and young adults, with an incidence of 3–4.5 cases per million annually (2, 3). Despite advances in surgical techniques and chemotherapy protocols, the prognosis for advanced-stage OS remains poor. While the 5-year survival rate reaches 60–70% in patients with localized tumors, it plummets to 10–20% for those with metastases or recurrence (4). These sobering statistics highlight the limitations of current standard therapies and underscore the urgent need for novel treatment strategies that address the aggressive biology and high metastatic potential of OS. The evolution of oncology from traditional cytotoxic modalities to modern targeted and immunotherapies provides a critical historical and clinical context for this challenge (5).

In recent years, increasing attention has been directed toward the intersection of cellular metabolism and immune regulation as a key determinant of cancer progression and therapeutic resistance. Studies published in 2019 and 2024 have demonstrated that metabolic rewiring—such as enhanced aerobic glycolysis and amino acid depletion—can impair antitumor immunity by generating immunosuppressive metabolites like lactate and kynurenine (6, 7). These metabolic byproducts alter the tumor microenvironment (TME), suppressing dendritic cell activation, T cell effector functions, and cytokine production (8, 9). The concept that metabolic alterations contribute to bone tumorigenesis can be traced back to 1978, when Smith et al. reported a case of metabolic bone disease resembling OS in a woolly monkey, triggered by calcium-phosphorus imbalance and vitamin D_3_ deficiency (10). Although this study did not directly explore immune mechanisms, it provided early evidence linking metabolic dysregulation to malignant bone lesions. Zhu et al. (2020) developed the first energy metabolism-related gene signature correlating with survival and immune infiltration in OS, representing highlighting a potential link between tumor metabolic status and clinical outcomes (11). This was followed by Zhang et al. (2021), who defined molecular subtypes of OS based on metabolic gene expression and demonstrated their association with immune cell profiles and prognosis (12).

Notably, OS is characterized by a metabolically active yet immunologically “cold” TME, in which dysregulated tumor metabolism not only sustains tumor growth but also imposes energetic and signaling constraints on infiltrating immune cells (13, 14). These features make immunometabolism a compelling framework for understanding how OS escapes immune surveillance and resists therapy. However, research specifically addressing immunometabolic mechanisms in OS remains scarce compared to other malignancies, limiting our understanding of how metabolic cues influence immune dynamics in this context.

**Figure 1:**
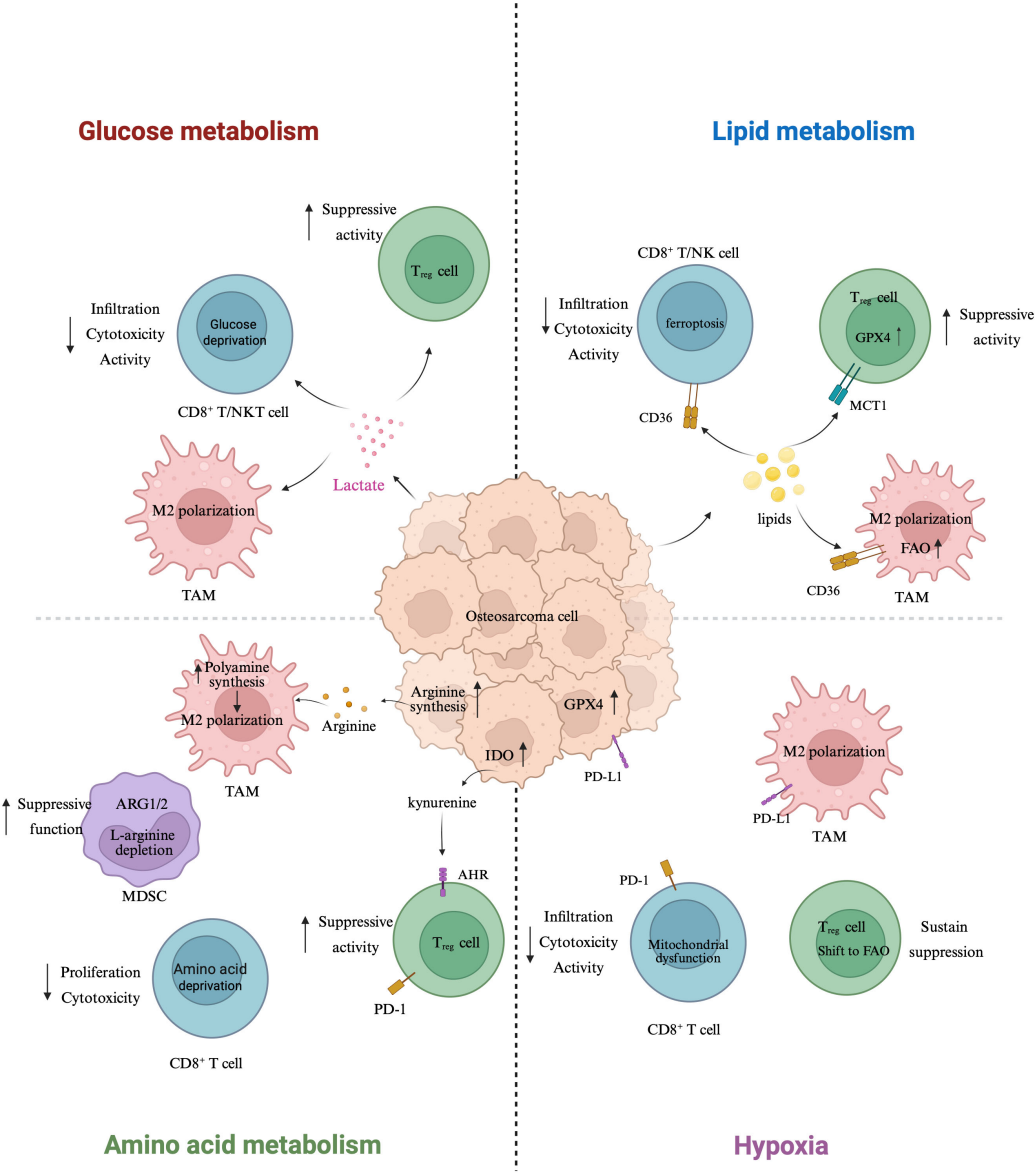
Immunometabolic remodeling in osteosarcoma: a four-axis schematic. Schematic overview of how metabolic rewiring in osteosarcoma (center) remodels immune function across four axes. Glucose metabolism (left-top): tumor-intrinsic aerobic glycolysis elevates lactate, which diffuses to immune cells, reduces CD8^+^ T/NK-cell infiltration and cytotoxicity, and favors Treg suppressive activity; lactate also supports M2-like polarization of tumor-associated macrophages (TAMs). Lipid metabolism (right-top): CD36-mediated lipid uptake in CD8^+^ T/NK cells promotes lipid peroxidation/ferroptosis, while GPX4 activity and MCT1-supported substrate use preserve Treg fitness; increased fatty-acid oxidation (FAO) in TAMs reinforces M2 programming. Amino-acid metabolism (left-bottom): ARG1/2-driven L-arginine depletion by MDSCs curtails CD8^+^ T-cell proliferation and function; tumor/host polyamine synthesis further skews TAMs toward M2 states; IDO-dependent kynurenine–AhR signaling expands Tregs and dampens antitumor responses. Hypoxia (right-bottom): hypoxia restriction induces mitochondrial dysfunction in CD8^+^ T cells and drives a Treg shift toward FAO, sustaining suppression. Tumor cells upregulate PD-L1, IDO, and GPX4 (center), collectively linking metabolic stress to checkpoint engagement and ferroptosis resistance. Arrows denote direction of influence; upward arrows reflect increased expression/activity. Created with BioRender.com.

To address this gap, the present review systematically integrates recent advances on metabolic reprogramming and its immunomodulatory effects in OS. In contrast to prior literature that either centers on single metabolic pathways or lacks an OS-specific perspective, we provide a comprehensive framework that unifies glucose, lipid, and amino acid metabolism with hypoxia-induced adaptations and their collective impact on the immune microenvironment. We further highlight translational opportunities based on emerging therapeutic strategies, aiming to inform future precision immunometabolic interventions for OS. To provide a conceptual roadmap for the sections that follow, we present an integrated schematic of immunometabolic remodeling in osteosarcoma, highlighting glucose, lipid, amino-acid, and hypoxia axes (**Figure 1**).

### 1.1 Literature search strategy

This review is based on a comprehensive literature search conducted using PubMed, Web of Science, and Scopus databases for articles published up to July 2025. Keywords included combinations of “osteosarcoma” AND “metabolism” OR “glycolysis” OR “lipid metabolism” OR “amino acid metabolism” OR “hypoxia” AND “immune microenvironment” OR “immunotherapy”. Additional manual screening of references from relevant articles was also performed. We included peer-reviewed original studies and reviews, prioritizing osteosarcoma-specific evidence. Non-English and non-peer-reviewed articles were excluded.

## 2 Metabolic reprogramming drives immunomodulatory remodeling in osteosarcoma

Metabolic rewiring in OS not only sustains tumor cell proliferation but also profoundly reshapes the immunological landscape of the TME. These metabolic alterations influence immune cell infiltration, function, and survival. Core metabolic pathways—including glucose, lipid, and amino acid metabolism—interact closely with immunoregulatory mechanisms to promote immune evasion and tumor progression. A summary of the major metabolic reprogramming pathways and their immunomodulatory effects in OS is provided in **Table 1**, with detailed discussions in Sections 2.1–2.3.

**Table 1:** Metabolic reprogramming pathways and their immunomodulatory effects in osteosarcoma.

Metabolic type	Reprogramming characteristics	Key molecules/enzymes	Impact on the immune microenvironment
Glucose Metabolism	Aerobic glycolysis (Warburg effect); oxidative PPP activation; PDK-mediated PDH inhibition → reduced mitochondrial oxidation; extracellular acidification	GLUT1, HK2, LDHA, G6PD, PDK	Lactate accumulation → PD-L1 upregulation via GPR81–TAZ; histone lactylation drives immunosuppressive gene programs; glucose competition → CD8^+^ T-cell mTORC1 suppression/functional exhaustion; Tregs utilize lactate via MCT1 to sustain suppressive activity
Lipid Metabolism	Enhanced lipid uptake, activation of *de novo* lipogenesis (DNL), lipid droplet storage, resistance to ferroptosis	CD36, FABP4, FASN, SCD1, DGAT1	TAM: CD36–PPARγ axis → M2-like polarization & survival; DC: lipid overload/XBP1 activation → impaired cross-presentation; CD8^+^ T cells: oxidized-lipid uptake (CD36) → lipid peroxidation/ferroptosis-like dysfunction; Tregs: increased FAO supports suppressive fitness
Amino Acid Metabolism	Activation of serine synthesis pathway, glutamine addiction, dysregulated branched-chain amino acid (BCAA) metabolism	PHGDH, GLS1, ANGPTL4, ODC1	Arginine depletion/ARG1/2 → impaired T-cell proliferation; polyamine accumulation (ODC1/AZIN1) → HLA-I downregulation & PD-L1 upregulation; Kynurenine–AhR signaling → Treg expansion & tolerogenic myeloid programs; GLN/LAT1/2–mTORC1 → macrophage phagocytosis escape (↑CD47); xCT–GPX4 maintains redox to evade ferroptosis; Methionine competition lowers T-cell SAM/epigenetic fitness

### 2.1 Glucose metabolic reprogramming in osteosarcoma immunometabolism

Under physiological conditions, healthy cells predominantly rely on mitochondrial oxidative phosphorylation for efficient adenosine triphosphate (ATP) production. In contrast, OS cells exhibit a hallmark metabolic shift toward aerobic glycolysis—commonly known as the Warburg effect—where glucose is preferentially converted to lactate even under normoxic conditions (15). This metabolic reprogramming not only meets the anabolic demands of rapid tumor proliferation but also enhances malignancy. Beyond fueling growth, glycolytic intermediates feed into biosynthetic pathways, while lactate and other byproducts actively remodel the TME by impairing effector immune cells, promoting immunosuppressive cell subsets, and facilitating immune evasion (16–18). A recent synthesis delineates lactic-acid metabolic reprogramming and metabolite-mediated communication in OS, consolidating evidence that lactate-rich niches orchestrate immune dysfunction and therapeutic resistance (19).

#### 2.1.1 Features of glucose metabolic reprogramming in osteosarcoma

The shift towards aerobic glycolysis in OS is an actively sustained oncogenic program, orchestrated by a network of key transcription factors. hypoxia-inducible factor-1α (HIF-1α) and the cellular MYC (c-MYC), which coordinately upregulate a suite of glycolytic genes, including glucose transporter 1 (GLUT1), hexokinase 2 (HK2), lactate dehydrogenase A (LDHA), enolase 1 (ENO1), and pyruvate kinase M (PKM) (20). These findings suggest that glycolysis in OS is not merely a metabolic byproduct but an actively maintained oncogenic program.

The clinical relevance of this glycolytic switch is powerfully underscored by the expression patterns of these enzymes. For instance, GLUT1 is overexpressed in 74.5% of OS tissues compared to only 11.8% in adjacent noncancerous tissues (21). High GLUT1 expression correlates with advanced TNM stage, lymph node metastasis, and poorer survival. In an separate cohort, GLUT1^+^ tumors (32.4%) were associated with markedly shorter disease-free survival and significantly lower microvessel density (22). These associations suggest a potential role for GLUT1 as a prognostic marker, although causality remains to be validated. This upregulation is driven by complex upstream signaling, with pathways such as P2RX7/c-Myc and USP22/β-catenin converging to enhance the transcription of GLUT1, HK2, and other key glycolytic genes in OS cells (23–25).

Beyond this core transcriptional axis, OS glycolysis is fine-tuned by a multi-layered regulatory network. In canine OS, STAT3 contributes to glycolytic reprogramming and invasion without immediate effects on proliferation (26). Additionally, circRNA Hsa_circ_0000566 enhances HIF-1α stability, thereby promoting GLUT1 and LDHA expression under hypoxic conditions (27); RNA modifications further enhance glycolytic transcript stability and translation: NAT10-mediated N^4^-acetylcytidine (ac^4^C) acetylation stabilizes PFKM and LDHA mRNAs (28), while METTL3-dependent N^6^-methyladenosine (m^6^A) modification of LINC00520—stabilized by USP13—supports ENO1 expression (29, 30). Pharmacological interrogation with compounds like Hydroxysafflor Yellow A (HYSA) has further confirmed the therapeutic targetability of the HIF-1α/HK2 axis in OS (31). Although promising, most of these studies remain at the preclinical stage, and their therapeutic relevance in OS patients warrants further exploration.

This intense glycolytic flux has profound downstream consequences, effectively decoupling glycolysis from mitochondrial oxidation and linking metabolism directly to epigenetic control. Pyruvate dehydrogenase kinase (PDK) activity increases, inhibiting pyruvate entry into the tricarboxylic acid (TCA) cycle. This forces pyruvate-to-lactate conversion, leading to extracellular acidification and the accumulation of lactate, which itself can serve as a substrate for histone lactylation—an epigenetic mark that alters gene expression. Concurrently, the buildup of certain TCA cycle intermediates, such as succinate and fumarate, can inhibit α-ketoglutarate–dependent demethylases, forging another direct link between metabolic state and epigenetic remodeling. A recent OS-specific study found that SIX4-mediated IDH1 upregulation enhances glycolysis–TCA flux and is associated with increased chromatin accessibility and therapy resistance (32).

#### 2.1.2 Glucose metabolism–mediated immunomodulation

In OS, enhanced glycolysis significantly alters the TME by promoting immune evasion via metabolic competition, immunosuppressive metabolite signaling, and stromal immune cell reprogramming. OS cells frequently overexpress GLUT1 and HK2, leading to excessive glucose uptake and limiting the availability of glucose for tumor-infiltrating CD8^+^ T cells. In such nutrient-restricted niches, T cells exhibit mTORC1 pathway inhibition, impaired oxidative phosphorylation, and reduced cytotoxic function (33–35).

A major immunoregulatory byproduct of this metabolic shift is lactate, which is far more than a terminal waste product. Lactate acidifies the TME and directly impairs CD8^+^ T-cell and NK cell function (36). While these effects have been robustly demonstrated in breast cancer and melanoma, their relevance in osteosarcoma remains underexplored. Lactate also promotes M2-like polarization of tumor-associated macrophages (TAMs) via the ERK/STAT3 signaling (37), and facilitates histone lactylation that upregulates expression of genes such as VEGFA and ARG1—mechanistically linking metabolic overflow to epigenetic immune remodeling (38). Furthermore, regulatory T cells (Tregs) can import lactate via monocarboxylate transporter 1 (MCT1) and utilize it oxidatively to sustain suppressive activity in lactate-rich niches (39–41). Although these findings are primarily derived from non-OS models, their conceptual relevance to the OS TME warrants focused investigation.

This theme of metabolic players exerting non-canonical, immunomodulatory functions extends to glycolytic enzymes themselves. While direct evidence in OS is still emerging, studies in other cancers provide compelling paradigms. In glioblastoma, the serine synthesis enzyme PHGDH, when expressed in endothelial cells, fuels aberrant angiogenesis and restricts T cell infiltration (42).

In liver cancer, nuclear PHGDH was shown to drive the production of chemokines that recruit immunosuppressive myeloid cells (43). These findings highlight a critical question: does PHGDH, which is known to be important in OS, play similar immunomodulatory roles in the bone TME?

Fortunately, emerging OS-specific research is beginning to anchor these general concepts in the context of bone sarcoma and uncover unique vulnerabilities. For instance, pharmacological inhibition of the glucose transporter SGLT2, which is robustly overexpressed in OS, was found to activate the cGAS-STING innate immune pathway, leading to enhanced CD8^+^ T cell infiltration and tumor suppression (44). Similarly, glucose restriction triggers the upregulation of NUCB2, a stress-adaptive factor that facilitates immune escape by stabilizing NUCKS1 and inducing the CXCL8–programmed death-ligand 1 (PD-L1) axis. Notably, NUCB2 knockdown synergizes with anti–PD-L1 therapy, leading to enhanced antitumor immune responses and tumor regression (45). These OS-specific mechanisms not only validate glucose metabolism as an immune checkpoint regulator in sarcoma but also provide rational targets for combinatorial immunotherapy.

Transcriptomic and integrative multi-omics analyses are increasingly employed to delineate immunometabolic subtypes of OS and identify predictive biomarkers.A recent study established a glycolysis-related four-gene risk signature—CHPF, RRAGD, TPR, and VCAN—which stratified OS patients based on immune infiltration, prognosis, and predicted drug response (46). In another large-scale analysis combining TARGET and GEO datasets, metabolism-based gene clusters were correlated with distinct immune microenvironment features. Within a vitamin and cofactor metabolism module, ST3GAL4 emerged as a key oncogene, promoting glycolysis and M2-like macrophage polarization. Knockdown of ST3GAL4 not only impaired glucose metabolism but also attenuated immunosuppressive TAM phenotypes *in vitro* and *in vivo* models (47). While these computational approaches offer valuable translational insights, their predictive robustness across independent clinical cohorts and functional validation in OS-specific models remain limited. Future studies should integrate multi-dimensional datasets with experimental validation to clarify causative relationships and guide personalized immunometabolic therapy.

In summary, while the immunomodulatory effects of aerobic glycolysis are potent, it is crucial to critically acknowledge that many of the detailed mechanisms described above have been primarily elucidated in non-sarcoma models. The OS research community must now move from plausible extrapolation to direct validation. Answering key questions—such as whether histone lactylation is a dominant epigenetic force in OS-associated TAMs or if the non-canonical functions of PHGDH are conserved in the bone TME—is paramount for developing truly effective immunometabolic therapies for osteosarcoma.

#### 2.1.3 Targeting glucose metabolism for osteosarcoma immunotherapy

Glycolytic reprogramming in OS is a key driver of immune evasion within the TME, providing a strong rationale for therapeutic intervention. Targeting glucose metabolism not only disrupts tumor bioenergetics but may also remodel the immunosuppressive TME to improve immunotherapy responsiveness. Current strategies can be broadly divided into direct inhibition of glycolytic enzymes and transporters, and targeting upstream regulatory hubs.

1. Inhibition of glucose uptake and glycolytic flux

Pharmacological inhibition of GLUT1 using WZB117 reduces glucose uptake and suppresses OS cell proliferation *in vitro* (48). While direct evidence of immune restoration in OS is lacking, studies in other cancers suggest that GLUT1 inhibition reverses M2-like macrophage polarization via the TGF-β1–Smad2/3 axis (49–51). These findings highlight GLUT1 as both a metabolic and immunologic regulator, although its immunomodulatory role in OS remains unvalidated.

2. Targeting lactate production and signaling

In OS, LDHA is a critical driver of lactate accumulation and tumor growth. Inhibition via FX11 or siRNA reduces lactate production, lowers extracellular acidity, and suppresses OS progression (52). However, the crucial question is whether this metabolic modulation can translate into enhanced immune responses. Here, the evidence requires careful interpretation. Although OS-specific proof that LDHA directly upregulates PD-L1 is not yet available, studies in lung and breast cancer models have shown that lactate upregulates PD-L1 via GPR81–TAZ signaling and impairs CD8^+^ T-cell function (53). Importantly, preclinical OS studies confirm that PD-1/PD-L1 blockade restores cytotoxic T lymphocyte (CTL) function and reduces metastases (54). Together, these findings suggest that combining glycolysis inhibition with checkpoint blockade may provide a rational therapeutic avenue in OS, but direct combinatorial evidence remains limited.

3. Upstream regulators and lineage programs

Several upstream molecular circuits maintain the glycolytic and immunosuppressive phenotype in OS:

- MicroRNA regulation: miR-328-3p directly targets GLUT1 in OS cells, lowering glucose uptake by roughly 30–50% and reducing lactate levels. Bioengineered miR-328-3p exhibits Chou–Talalay synergy with cisplatin or doxorubicin in OS cells (55).
- Circular RNA–miRNA axis: circ_0004674 promotes expression of GLUT1, HK2, PKM2, and LDHA via the miR-140-3p/TCF4 axis, enhancing glycolysis and invasive behavior. Silencing circ_0004674 inhibits OS cell migration and invasion (56).
- P4HA1 as a glycolysis–immunity node: Within glycolysis-related prognostic gene sets, P4HA1 is upregulated in OS and promotes proliferation in a glycolysis-dependent manner. Inhibition via 2-deoxy-D-glucose (2-DG) attenuates this effect (57). Intriguingly, in non-OS tumor models, P4HA1 suppression expands TCF1^+^ CD8^+^ progenitor pools and reduces exhaustion, suggesting a potential link between glycolytic flux and T-cell fate regulation (58).

These insights underscore the complexity of targeting OS metabolism—where metabolic, epigenetic, and immune programs are tightly interwoven.

### 2.2 Lipid metabolic reprogramming in osteosarcoma immunometabolism

Beyond glycolysis, OS cells extensively rewire their lipid metabolism to serve two primary functions: securing a flexible fuel source for bioenergetics and constructing a robust defense against oxidative stress, particularly ferroptosis. This reprogramming involves a coordinated upregulation of lipid synthesis, uptake, and storage, creating a lipid-rich phenotype that profoundly shapes both tumor progression and its interaction with the immune system.

#### 2.2.1 Features of lipid metabolic reprogramming in osteosarcoma

OS cells retune their metabolic machinery to maximize lipid availability. Key OS-specific adaptations include:

- Enhanced *De Novo* Lipogenesis: At the transcriptional level, sterol regulatory element–binding protein 1 (SREBP-1), activated by PI3K/AKT signaling, upregulates fatty acid synthase (FASN) (59), which catalyzes palmitate synthesis and promotes OS progression partly through the HER2/PI3K/AKT axis (60).
- Fatty Acid Oxidation (FAO) as an Energy Source: To meet their high energy demands, OS cells utilize FAO, catalyzed by enzymes like CPT1A, to feed acetyl-CoA into the TCA cycle (61), while disruption of long-chain fatty acid β-oxidation in a murine S-180 OS model reduces ATP availability and impairs tumor viability (62).
- Increased Lipid Uptake: Tumor cells enhance exogenous lipid acquisition by upregulating CD36, fatty acid transport proteins (FATPs), and fatty acid–binding proteins (FABPs) (63–65). In OS, FABP4 expression can be induced by the lipid metabolism–associated lncRNA RPARP-AS1, which also upregulates MAGL and stearoyl-CoA desaturase 1 (SCD1), potentially through the Akt/mTOR pathway (66).
- Lipid droplet (LD) accumulation: Excess lipids are stored in LDs mainly via DGAT1, with possible contribution from ACAT, to prevent lipotoxicity (67). Transcriptomic profiling has revealed two lipid metabolic OS subtypes: a lipid-anabolic cluster enriched in cholesterol and fatty acid synthesis with poor prognosis, and a PUFA/steroid-enriched cluster associated with better outcomes (68). This metabolic heterogeneity highlights the prognostic significance of lipid programs in OS, though their direct contributions to tumor progression remain incompletely defined.

A central feature of this lipid reprogramming is the establishment of a powerful anti-ferroptotic defense. Ferroptosis, a form of iron-dependent cell death driven by lipid peroxidation, represents a key vulnerability for cancer cells. OS cells counter this threat by meticulously controlling their lipid composition. A pivotal enzyme in this process is stearoyl-CoA desaturase (SCD), transcriptionally driven by c-Myc in OS, converts saturated fatty acids into monounsaturated fatty acids (MUFAs), mitigating lipid peroxidation; its pharmacological inhibition triggers ferroptosis *in vitro* and *in vivo*, underscoring its therapeutic potential (69). This intricate metabolic network is not without its complexities. The enzyme ACSL4, for example, presents a therapeutic paradox: while its activity is required to generate the PUFA-containing lipids that are substrates for ferroptosis, it has also been shown to promote OS progression via TGF-β signaling (70). This paradox underscores the therapeutic challenge of targeting ACSL4, as selective modulation of its pro-tumorigenic functions without undermining ferroptosis sensitivity remains unresolved.

Beyond intrinsic tumor adaptations, extrinsic metabolic regulation also contributes. M2 macrophage–derived exosomes deliver apolipoprotein C1 (Apoc1) to OS cells, where Apoc1 interacts with ACSF2 and prevents its deubiquitination by USP40, leading to ACSF2 degradation and suppression of ferroptotic death (71). This highlights sophisticated metabolic crosstalk whereby immune cells actively shield tumor cells from ferroptotic stress. More broadly, extracellular vesicles serve as central mediators of metabolic reprogramming within the tumor microenvironment, shaping intercellular communication and tumor progression (72).

#### 2.2.2 Lipid metabolism–mediated immunomodulation

The profound rewiring of lipid metabolism in OS not only fuels tumor growth but also reshapes the TME into a lipid-saturated niche that actively suppresses antitumor immunity. While some immunomodulatory effects have been validated in OS-specific studies, others are extrapolated from carcinoma models and require further confirmation in sarcoma contexts.

- Polarizing TAMs: The lipid-rich environment is readily exploited by TAMs. Through transporters like CD36, TAMs increase their lipid uptake and storage in lipid droplets. This accumulated lipid fuels their FAO, a metabolic program strongly associated with the immunosuppressive M2-like phenotype (73, 74).. This metabolic state is reinforced by PPARγ-mediated transcription of lipid metabolic genes and reshaping of endoplasmic reticulum (ER) membrane lipids (75, 76).
- Paralyzing Dendritic Cells (DCs): In non-OS models, excessive lipid accumulation in DCs has been shown to be detrimental, impairing their ability to process and present antigens, thereby weakening the priming of naïve T cells. This dysfunction is often exacerbated by ER stress and the activation of the XBP1 pathway (77, 78). Whether this mechanism is a major contributor to immune evasion in the unique bone TME of osteosarcoma remains an important open question.
- Driving T-Cell Ferroptosis and Exhaustion: Perhaps the most critical consequence of lipid dysregulation is its direct impact on T cells.
- CD8^+^ T Cells: Infiltrating CD8^+^ T cells are particularly vulnerable. The uptake of oxidized lipids via CD36 can trigger overwhelming lipid peroxidation, culminating in ferroptosis and the loss of effector function. This concept, primarily established in melanoma models, suggests that the very lipids fueling the tumor are toxic to the cells meant to destroy it. Interrupting this axis restores T-cell function and synergizes with anti–programmed cell death protein 1 (PD-1) therapy, pointing to a key metabolic checkpoint (79).
- Tregs cells: In contrast, Tregs appear to be more resilient to this lipid stress (80). They are shielded from ferroptosis by high expression of the antioxidant enzyme glutathione peroxidase 4 (GPX4). This differential sensitivity is therapeutically intriguing: inducing a controlled level of ferroptotic stress might selectively eliminate Tregs while sparing effector T cells, thus tipping the immune balance in favor of an antitumor response (81, 82).

In summary, the rewired lipid metabolism of OS establishes an immunosuppressive TME through multiple, interconnected mechanisms. However, it is imperative to underscore that many of these elegant immunomodulatory mechanisms, particularly those involving T-cell ferroptosis, have been elucidated in carcinomas. Validating their significance in the sarcoma context is a critical priority for the field.

#### 2.2.3 Targeting lipid metabolism for osteosarcoma immunotherapy

Collectively, the immunosuppressive effects of lipid metabolic reprogramming in OS are mediated through altered fatty acid utilization, lipid peroxidation, and ferroptosis regulation across multiple immune subsets. Building on these mechanisms, targeted interventions in lipid metabolism may simultaneously disrupt tumor metabolic dependencies and restore effective immunity.

1. Blocking lipid synthesis or uptake

- Targeting FASN: Targeting the key lipogenic enzyme FASN has been shown to suppress OS growth in preclinical models. This can be achieved not only through direct inhibitors but also with agents such as Brusatol, which rewires PI3K/AKT and MAPK signaling to reduce FASN expression in OS cells (83). The immunomodulatory rationale is even more compelling, though it remains speculative for OS. In hepatocellular carcinoma, FASN blockade was found to increase MHC-I antigen presentation on tumor cells, enhancing their recognition by CD8^+^ T cells and synergizing with PD-1/PD-L1 blockade (84). Validating whether this crucial mechanism is conserved in OS is a key future task.
- Targeting CD36: Blocking the lipid transporter CD36 offers another strategy. In non-OS cancer models, CD36 blockade was shown to protect intratumoral CD8^+^ T cells from lipid-induced ferroptosis, restoring their effector function and improving the efficacy of anti–PD-1 therapy (79). This positions CD36 as a high-priority target for investigation in OS immunomodulation.

2. Triggering ferroptosis to sensitize immunotherapy.

Given that OS cells devote substantial resources to evading ferroptosis, an effective countermeasure is to drive them into this cell-death program. Moreover, selective autophagy—including ferritinophagy, lipophagy, mitophagy, and chaperone-mediated autophagy—acts as an upstream regulatory hub of ferroptosis, providing druggable entry points to harness autophagy–ferroptosis crosstalk for immunometabolic modulation (85). A growing body of preclinical work in OS has identified multiple ways to achieve this:

- Targeting the Central GPX4/xCT Axis : Several agents, including the natural compound baicalin, can induce ferroptosis in OS cells by downregulating the core anti-ferroptotic machinery components GPX4 and xCT (86). Mechanistically, lncRNA PVT1 activates the STAT3/GPX4 axis to suppress ferroptotic lipid peroxidation and drive OS progression, highlighting an actionable node for restoring ferroptosis sensitivity (87).
- Advanced Delivery Systems: Innovative approaches, such as exosomal delivery of miR-144-3p (88) or nanoparticles co-delivering cisplatin and ferroptosis inducers (89), have shown synergistic efficacy in OS models, successfully combining ferroptosis with chemosensitization.

Crucially, while these strategies effectively induce ferroptosis and enhance chemosensitivity in OS, the next vital step is to determine if they can similarly sensitize OS to immune checkpoint blockade. The principle is well-supported by non-OS models, where inducing ferroptosis was shown to potentiate anti–PD-1 responses (90). Bridging this concept to OS-specific models represents a major therapeutic opportunity.

3. Combination strategies and resistance reversal.

Beyond direct induction, more advanced strategies are emerging. Dihydroartemisinin synergizes with VEGFR TKIs by disrupting lipid pathways and attenuating LOXL2-mediated VEGFA expression, overcoming antiangiogenic resistance (91). Differentiation therapy coupled with ROS-amplified ferroptosis suppresses OS progression and targets stem-like populations, pointing to a route for tackling chemoresistance (92).

### 2.3 Amino acid metabolic reprogramming in osteosarcoma immunometabolism

Amino acid metabolism in osteosarcoma is comprehensively rewired to satisfy the diverse demands of a malignant cell. This reprogramming extends far beyond simply providing building blocks for protein synthesis; it is crucial for fueling biosynthesis, maintaining redox homeostasis, and executing a robust defense against metabolic stress and cell death pathways like ferroptosis.

#### 2.3.1 Features of amino acid metabolic reprogramming in osteosarcoma

We can understand the reprogramming of amino acid metabolism in OS by grouping the adaptations according to their primary function:

- Fueling Biosynthesis and Anaplerosis: OS cells extensively reprogram amino acid metabolism to sustain proliferation, maintain redox balance, and support biosynthesis. In the serine synthesis pathway, phosphoglycerate dehydrogenase (PHGDH) diverts glycolytic flux toward serine production, enabling nucleotide synthesis, NADPH generation, and glutathione biosynthesis (93). PHGDH is upregulated in more than 50% of OS tumors, sustained by mTORC1–ATF4 signaling, and its high expression predicts poor relapse-free survival (HR = 1.93) and overall survival (HR = 1.86) (94). Similarly, OS cells often display glutamine addiction, relying on the enzyme glutaminase (GLS) to convert glutamine into α-ketoglutarate, which replenishes the TCA cycle (anaplerosis) and supports redox balance (95). High GLS1 expression also correlates with poor prognosis in OS patients (96).
- Regulating Oncogenic Signaling: Amino acid levels can also directly influence intracellular signaling. The role of branched-chain amino acids (BCAAs) appears complex and context-dependent. While exogenous leucine can promote OS growth by activating the mTORC1 pathway, the regulation of intracellular BCAAs by ANGPTL4 presents a more complicated picture. One study reported that ANGPTL4 acts as a tumor suppressor by restraining intracellular BCAA levels, thereby preventing mTORC1 hyperactivation (97). By contrast, other studies have found that increased ANGPTL4 promotes OS proliferation, osteoclastogenesis and angiogenesis—e.g., via the CCAL–miR-29b–ANGPTL4 axis—highlighting potential context-dependent effects (98, 99). These apparently opposing roles indicate that ANGPTL4 may act as a metabolic rheostat whose impact depends on genetic or micro-environmental context. In line with this, exogenous leucine accelerates tumor growth through AMPK suppression and mTORC1 activation (100).
- Maintaining Redox Balance and Evading Ferroptosis: A central defensive strategy for OS cells is the upregulation of the cystine-glutathione axis to combat oxidative stress and ferroptosis. Upregulation of xCT (SLC7A11) increases cystine import for glutathione synthesis, which supports GPX4-mediated detoxification of lipid peroxides. In human OS cell lines, the transcription factor MLX directly enhances SLC7A11 expression. MLX knockout reduces SLC7A11 levels, depletes glutathione, elevates ROS, and induces ferroptosis—all reversible by SLC7A11 overexpression (101). Similarly, Similarly, PSAT1 depletion suppresses xCT and GPX4 expression, leading to oxidative stress and ferroptotic death, which can be reversed by Ferrostatin-1 (102). These results establish the xCT–GPX4 axis as a metabolic checkpoint linking redox defense to cell survival.
- Adapting to Nutrient Scarcity: In the nutrient-poor TME, OS cells must be able to synthesize their own resources. In OS, NUCKS1 upregulates asparagine synthetase (ASNS), supporting protein synthesis and mitochondrial function. Silencing NUCKS1 impairs tumor growth and migration *in vitro* and *in vivo* (103). Although direct immunologic consequences in OS remain to be tested, in other cancers, elevated asparagine enhances N-glycosylation of immunoregulatory proteins such as PD-L1—a potential intersection of metabolic and immune regulation that warrants further investigation.

#### 2.3.2 Amino acid metabolism–mediated immunomodulation

The dysregulated amino acid metabolism of OS cells creates a metabolically hostile TME that actively sabotages antitumor immunity. These effects are mediated through nutrient competition, the secretion of immunosuppressive catabolites, and the modulation of immune evasion pathways. However, it is critical to note that many of the following mechanisms have been primarily defined in non-OS cancer models and represent compelling, yet largely unproven, hypotheses in the context of osteosarcoma.

• Arginine Depletion and Polyamine Production: The depletion of L-arginine from the TME is a classic mechanism of immune suppression. This is often carried out by myeloid-derived suppressor cells (MDSCs), which express high levels of arginase (ARG1), starving T cells of an amino acid essential for their proliferation and function (104). Within OS cells themselves, the AZIN1 enzyme shunts arginine towards polyamine synthesis. This not only fuels tumor proliferation but has also been shown in OS models to suppress CD8^+^ T cell cytotoxicity and reduce MHC-I expression (105). Furthermore, in other cancer models, tumor-derived arginine can fuel polyamine production in TAMs, locking them into an M2-like state (106). A polyamine metabolism–related gene signature also stratifies OS prognosis and correlates with poor immune infiltration (107).

• Modulating “Don’t Eat Me” and Immune Checkpoint Signals:

  • The LAT2–CD47 Axis:

The neutral amino acid transporter LAT2 (SLC7A8) has been shown in other cancers to activate mTORC1 signaling, leading to upregulation of the “don’t eat me” signal CD47, thus protecting tumor cells from macrophage phagocytosis (108). In OS cohorts, a locus in SLC7A8 is associated with early disease progression, and LAT2 functions as a transporter for doxorubicin; notably, low LAT2 expression in non-metastatic patients correlates with poorer survival (109), this specific immunomodulatory function has not yet been verified in osteosarcoma.

  • The ASNS–PD-L1 Hypothesis:

The upregulation of ASNS, driven by NUCKS1 in OS, supports metabolic adaptation. It is hypothesized that increased asparagine levels may enhance the N-linked glycosylation of PD-L1, stabilizing it on the tumor cell surface and prolonging its immunosuppressive effects (103). However, direct evidence for this mechanism in OS remains lacking.

• Tryptophan Catabolism—A Minor Pathway in OS?: The indoleamine 2,3-dioxygenase 1 (IDO1)–kynurenine–AhR axis is a dominant immunosuppressive pathway in many cancers such as melanoma. The enzyme IDO1 degrades tryptophan into kynurenine, which activates the AhR receptor in T cells, promoting Treg differentiation and T cell exhaustion (110, 111). However, a crucial finding is that IDO1 expression was detected in 6.71% of primary OS tumors over a 10-year cohort (112). This strongly suggests that, unlike in other immunogenic tumors, tryptophan catabolism is not a primary mechanism of immune escape for the majority of OS patients—a critical consideration when designing immunotherapy strategies.

• Epigenetic Reprogramming via One-Carbon Metabolism:

Amino acids such as methionine and serine feed into one-carbon metabolism to generate S-adenosylmethionine (SAM), a key methyl donor for histone methylation. In non-OS models, tumor competition for methionine depletes SAM in T cells, impairing H3K79me2 and STAT5 signaling and ultimately reducing effector functions (113). In macrophages, methionine uptake supports SAM-dependent H3K36me3 (114). In Tregs, glutathione constrains serine uptake to maintain low mTOR signaling and FoxP3 stability; limiting serine/glycine availability can rescue suppressive capacity under glutathione-deficient conditions (115). These findings hint at unexplored epigenetic–metabolic crosstalk in OS.

• Immunoregulatory catabolites: Catabolite accumulation can reprogram T cells, as glutarate inhibits TET2/KDM demethylases and glutarylates PDHE2, enhancing glycolysis and promoting CD8^+^ memory differentiation with improved antitumor cytotoxicity (116). In non-OS models, Slc3a2-mediated BCAA uptake sustains mTORC1 and Treg suppressive function (117), though whether this applies to OS remains untested.

Clinical correlates support these mechanisms. Soluble immune checkpoints—including sIDO, sTIM3, sCTLA4, and sCD137—are associated with metastasis risk and poor survival in OS patients (118). Conversely, a high intratumoral CD8^+^/FOXP3^+^ ratio (>3.08) predicts significantly improved overall survival over a median follow-up of 69 months (119), reinforcing the prognostic impact of T-cell metabolic fitness.

#### 2.3.3 Targeting amino acid metabolism for osteosarcoma immunotherapy

Given the diverse roles of amino acids in both tumor growth and immune suppression, targeting these pathways presents a multifaceted therapeutic opportunity. Strategies can be broadly divided into those targeting tumor-intrinsic dependencies and those aimed at remodeling the immune microenvironment.

(1) Exploiting Tumor-Intrinsic Metabolic Addictions. The dependencies of OS on specific amino acid pathways reveal actionable vulnerabilities.

• Targeting Serine and Glutamine Metabolism:

Inhibitors of PHGDH and GLS1 have shown preclinical efficacy in suppressing OS growth (94, 120). However, a significant challenge is metabolic compensation; inhibiting PHGDH, for instance, can trigger pro-survival mTORC1 signaling as a compensatory response. This suggests that effective treatment will likely require co-inhibition strategies, such as combining PHGDH inhibitors with mTORC1 or AKT inhibitors, which has shown strong synergy in OS models (121).

• Disrupting Metastasis-Linked Epigenetic Drivers:

Ailanthone disrupts the KMT2A–MEN1 complex, suppressing serine synthesis pathway (SSP) genes and lung metastasis (122).

• Overcoming Chemoresistance via Metabolic Rewiring:

RFWD3 promotes chemoresistance by ubiquitin-mediated degradation of PHGDH, conserving cellular NAD^+^ and driving *de novo* nucleotide biosynthesis; lomitapide disrupts the RFWD3–PHGDH axis and reverses this resistance (123).

• Exploiting Synthetic Lethality:

The addiction of some OS subtypes to glutamine can be exploited through synthetic lethality. For example, in OS models driven by the YAP1 oncogene, inhibiting glutaminolysis with a GLS1 inhibitor creates a dependency that can be lethally targeted by inhibiting polyamine synthesis with the FDA-approved drug DFMO (124). The RPS27–RPS24 fusion promotes glutaminolysis and chemoresistance via cuproptosis suppression in OS (125).

(2) Remodeling the Immune Microenvironment.

• Arginine Depletion and Modulation:

Given that arginine depletion is typically immunosuppressive, a counterintuitive but potentially effective strategy tested in SIRPA-overexpressing OS xenografts was the use of recombinant arginase to further deplete systemic arginine. This approach curtailed metastasis, possibly by disrupting an arginine-uptake loop required by the tumor cells themselves, namely the SIRPA–SP1–SLC7A3 axis (126).

• Nanoparticle Co-delivery with IDO Inhibition:

Nanoparticles co-delivering IDO inhibitors with platinum drugs activate cGAS–STING signaling, increase DNA damage, and enhance CD8^+^ T-cell infiltration in OS (127).

• Serine/Glycine Restriction:

Dietary interventions, such as restricting serine and glycine, have been shown in other cancer models to boost CD8^+^ T-cell activity. However, this approach is a double-edged sword, as it can also paradoxically increase immune evasion by promoting the lactylation and stabilization of PD-L1. This highlights the complexity of metabolic interventions and underscores the need for combining dietary modulation with PD-1 blockade to achieve a net antitumor effect (128).

Importantly, these dependencies can be noninvasively monitored using PET tracers. In MG63.3 xenografts, GLS1 inhibition by CB-839 altered glutamine uptake and metabolic flux, inducing a transient [^18^F]FLT “flare effect” reflecting a post-treatment proliferative rebound (129).

## 3 Hypoxia: a master regulator amplifying immunometabolic suppression

Hypoxia, a pervasive feature of the poorly vascularized OS microenvironment, is not merely another stress factor but a master regulator that dramatically amplifies the metabolic and immunosuppressive programs previously discussed. The stabilization of hypoxia-inducible factors (HIFs), primarily HIF-1α and HIF-2α, acts as a central command node. It intensifies the Warburg effect, reshapes lipid and amino acid utilization for survival, and orchestrates a multi-faceted assault on antitumor immunity, thus creating a uniquely challenging therapeutic target. **Table 2** outlines hypoxia-induced metabolic programs and their immune consequences.

**Table 2:** Hypoxia-induced metabolic and immune remodeling in the OS microenvironment.

Hypoxic feature	Metabolic reprogramming mechanism	Immunoregulatory effect	Key molecules
Oxygen diffusion barrier caused by mineralized matrix	HIF-1α stabilization; P4HA1-driven collagen modification → denser ECM and aggravated hypoxia	Myeloid skewing toward M2-like programs and PD-L1 upregulation; CD8^+^ T-cell mitochondrial dysfunction and metabolic exhaustion	HIF-1α, P4HA1,
Metabolic symbiosis between hypoxic and oxygenated zones	Glycolysis-to-lactate with LDHA/PDK1 in hypoxic cells; MCT4→MCT1 lactate shuttle conserves glucose; mitochondrial SLC1A5 variant and SLC25A15 sustain glutamine anaplerosis/redox	Lactate signaling via GPR81–TAZ drives PD-L1 induction; nutrient sparing complicates therapy and sustains immunosuppression	LDHA, PDK1, MCT4, MCT1, GPR81, TAZ, SLC1A5, SLC25A15
Acidic microenvironment (pH 6.5–6.8)	CA9-mediated proton extrusion →maintenance of intracellular alkalization	Inhibition of effector T cell activity, promotion of MDSC function	CA9, HCO_3_^-^

### 3.1 Hypoxia-induced metabolic rewiring in osteosarcoma

Under hypoxic conditions, OS cells undergo an intensified metabolic reprogramming to adapt and survive.

• Supercharging Glycolysis: Hypoxia is the most potent activator of the Warburg effect. HIF-1α directly drives the overexpression of nearly all key glycolytic machinery, including the transporters GLUT1 and enzymes HK2 and LDHA. Simultaneously, it upregulates PDK1, which shunts pyruvate away from the mitochondria, cementing the cell’s reliance on lactate production (31, 130). This creates pockets of intense extracellular acidification, which are managed by pH regulators like CA9 (131). Meanwhile, an MCT4→MCT1 lactate shuttle couples hypoxic and oxygenated zones, creating metabolic symbiosis that conserves glucose and complicates treatment (132, 133), disrupting either transporter might break this cooperation and resensitize tumor to therapy.

• Reshaping Lipid and Amino Acid Metabolism:

Hypoxia reshapes lipid use as well. HIF-2α promotes lipid droplet programs and restrains lipolysis/FAO, driving LD accumulation as stress buffering (134). Cross-tumor data show a HIF-2α–LPCAT1–FBXW7 axis that degrades ACLY and rewires membrane lipid composition (135); whether this operates in OS remains to be tested. Amino-acid handling is also rewired: a mitochondrial SLC1A5 variant boosts glutamine anaplerosis, and hypoxia-responsive SLC25A15 supports redox balance (136, 137).

### 3.2 Hypoxia-driven remodeling of the immune microenvironment

• Skewing Myeloid Cells:

Hypoxia is a powerful signal that polarizes myeloid cells toward an immunosuppressive, pro-tumor M2-like phenotype. In various cancer models, HIF-1α stabilization in macrophages and MDSCs has been shown to directly drive the expression of PD-L1 (138, 139). Together, these loops connect metabolic acidification to checkpoint up-regulation, rationalizing combined lactate- and PD-L1-targeted therapy.

• Exacerbating T-Cell Dysfunction:

For lymphocytes, For lymphocytes attempting to infiltrate the tumor, the hypoxic TME is exceptionally hostile. Hypoxia impairs mitochondrial function in CD8^+^ T cells, driving them toward metabolic exhaustion. Work in non-OS models has shown that the collagen-modifying enzyme P4HA1, itself induced by hypoxia, can disrupt α-KG metabolism and limit the expansion of effective progenitor T-cell populations, while P4HA1 inhibition restores mitochondrial fitness and enhances antitumor CD8^+^ responses (58). In contrast, Tregs are well-adapted to thrive in hypoxic, lactate-rich niches by shifting their metabolism toward FAO (140). Additionally, tumor-derived lactate promotes Treg proliferation and immunosuppression via MOESIN lactylation and TGF-β signaling enhancement (141).

• Impairing Antigen Presentation:

DCs function is also crippled by hypoxia. Studies in other contexts have revealed that the CCR7-inducible lncRNA lnc-Dpf3 antagonizes HIF-1α, limiting glycolytic reprogramming and DC migration and activation, thereby weakening antitumor T-cell priming (142).

• Bolstering Tumor Defenses:

Hypoxia also elevates resistance to ferroptotic death by upregulating SLC7A11/GPX4, suppressing lipid peroxidation and potentially reducing sensitivity to IFN-γ–mediated tumor killing (see §§2.2.2, 2.3.2).

### 3.3 Therapeutic implications and future perspectives

• Direct HIF pathway inhibition:

HIF-2α inhibitors show dual immune–metabolic effects across tumors: belzutifan + cabozantinib achieved a 70% ORR in LITESPARK-003 (ccRCC); LITESPARK-005 showed better patient-reported outcomes *vs* everolimus (143). However, given the absence of VHL mutations in OS, extending its use to this context requires biomarker-driven strategies to identify HIF-2α–dependent tumors.

• PHD Modulation:

A paradoxical but promising approach involves using PHD inhibitors like roxadustat to induce a “pseudohypoxic” state in T cells. In microsatellite-stable colorectal cancer models, this strategy enhanced T-cell function and boosted anti–PD-1 efficacy (144). Whether this can be extrapolated to OS remains to be validated.

• Hypoxia-activated prodrugs:

The hypoxia-activated prodrug TH-302 targets is more potent against OS xenografts when paired with pro-apoptotic receptor agonists, and combination data from other sarcomas support adding chemotherapy, anti-angiogenics, or radiation (145). Additional sarcoma data support its combination with chemotherapy, angiogenesis inhibitors, or radiation, justifying further exploration in OS-specific trials.

## 4 Clinical translation and therapeutic opportunities

While preclinical studies have illuminated a rich landscape of metabolic vulnerabilities in OS, the path to successful clinical translation is fraught with immense challenges. The transition from promising data in homogenous cell lines and animal models to meaningful efficacy in heterogeneous patient populations has been slow across all of oncology, and OS is no exception. A successful translational strategy requires not only potent inhibitors but also a deep understanding of combination therapies, advanced delivery systems, and, most critically, robust predictive biomarkers to guide their use. This section will critically evaluate the most promising therapeutic strategies and outline a framework for their future clinical development. **Table 3** lists metabolism-targeted therapeutic approaches, mechanisms, and supporting evidence.

**Table 3:** Targeting tumor metabolism in osteosarcoma: therapeutic approaches and experimental validation.

Therapeutic target	Intervention drugs/Strategies	Mechanism of action	Preclinical/Clinical evidence
Lipid Metabolism	FASN inhibitors (TVB2640, cerulenin); SCD1 inhibitors (e.g., MF-438/CAY10566)	Block *de novo* palmitate synthesis; reduce MUFA, increase lipid peroxidation; downregulate HER2/PI3K/AKT; sensitize tumors to ferroptosis	OS preclinical: tumor growth/metastasis reduced; Clinical (cross-tumor): TVB-2640 in early-phase trials; OS-specific clinical data pending
Glucose Metabolism	GLUT1 inhibitor (WZB117); LDHA inhibitors; PDK inhibitors (e.g., DCA class)	Decrease glycolytic flux and lactate output → mitigate extracellular acidification; improve CD8^+^ T-cell infiltration and DC function; potential synergy with anti–PD-1/PD-L1 and radiotherapy	OS preclinical: improved T-cell infiltration and checkpoint blockade synergy reported; Clinical: target-class activity in other tumors; OS trials needed
Amino Acid Metabolism	DFMO (ODC1 inhibitor; polyamine blockade); Methionine depletion (enzyme-based/microbial); ± GLS1 inhibitors	Polyamine depletion may restore HLA-I and limit PD-L1; methionine restriction reduces 1-carbon/SAM flux and proliferation; intermittent schedules to spare T cells	OS preclinical: DFMO reduces tumor burden; methionine restriction suppresses growth/metastasis; Combinations with checkpoint blockade show enhanced efficacy in murine models
Hypoxic Microenvironment	HIF-2α antagonists (e.g., belzutifan; PT2399 as tool compound); CA9 inhibitors (e.g., SLC-0111)	Remodel hypoxia-driven immune–metabolic programs; reduce extracellular acidification and improve effector T-cell function	Clinical (cross-tumor): belzutifan-based regimens active in RCC; OS: direct clinical data limited—biomarker-guided trials warranted

### 4.1 Direct inhibition of key metabolic nodes

The most straightforward approach involves targeting the key enzymes that fuel OS metabolism.

• Targeting Lipid Synthesis:

As discussed, OS cells often exhibit a dependency on *de novo* lipogenesis. The FASN inhibitor TVB-2640 has entered early-phase clinical trials for various solid tumors (ClinicalTrials.gov Identifier: NCT02223247). While OS-specific clinical data is absent, the strong preclinical rationale—including evidence that genetic or pharmacologic FASN blockade suppresses growth and invasion and downregulates HER2/PI3K/AKT signaling in OS models—makes this an attractive avenue for future investigation (60, 146). A similar rationale applies to SCD1, whose inhibition effectively triggers ferroptosis in preclinical OS models and warrants further exploration.

• Targeting Amino Acid Metabolism:

The dependency of some OS subtypes on polyamine and glutamine metabolism has led to promising preclinical results with the ornithine decarboxylase 1 (ODC1) inhibitor DFMO, particularly in synthetic lethal combinations that exploit YAP1-mediated glutamine addiction (124). Likewise, preclinical OS models have shown sensitivity to methionine restriction, a strategy now being explored through enzymatic or microbial depletion methods (147, 148).

### 4.2 The imperative of combination therapy

Given the metabolic plasticity of OS, it is widely accepted that monotherapy with metabolic inhibitors is unlikely to be curative. Their true potential lies in their ability to sensitize tumors to other therapeutic modalities, particularly immunotherapy.

The central hypothesis is that metabolic inhibitors can remodel the hostile TME into a more immune-permissive state. Strategies aimed at reducing lactate production (e.g., via LDHA or MCT4 inhibitors) or remodeling the hypoxic microenvironment (e.g., via HIF inhibitors) are prime examples. By alleviating the key immunosuppressive signals discussed in Sections 2 and 3, these agents could theoretically unleash the full potential of immune checkpoint inhibitors. A recent OS-specific study elegantly demonstrated this principle using a multifunctional CaCO_3_-based nanoplatform that simultaneously neutralized tumor acidity and delivered a lactate-suppressing agent, resulting in enhanced CD8^+^ T cell infiltration and improved efficacy of PD-1 blockade (149). This highlights the potential of nanotherapeutic strategies to co-target metabolic and immune axes in OS.

### 4.3 Advanced delivery systems for precision and safety

A major hurdle for systemic metabolic inhibitors is the potential for on-target toxicity in healthy, metabolically active tissues (including immune cells). Advanced delivery systems are therefore not just an enhancement but a potential necessity for clinical success. Bone-targeted delivery systems—such as osteoprogenitor cell–mediated liposomal delivery and rationally designed lipid or polymeric nanocarriers—can enhance drug accumulation in skeletal lesions and improve antitumor activity in OS models while minimizing off-target toxicity (150, 151). Beyond biodistribution control, lipid-based nanoparticles can interface with macrophages to tune tissue homing, payload release, and immune interactions, providing a generalizable delivery framework for immunometabolic interventions (152). In parallel, iron-based coordination assemblies have been engineered for *in vivo* diagnosis and therapy, offering a tunable platform to amplify oxidative stress and potentially augment ferroptosis-oriented regimens (153).

### 4.4 Predictive biomarkers and prognostic models

Perhaps the single greatest barrier to the clinical translation of metabolic therapies is the lack of validated predictive biomarkers. OS is a highly heterogeneous disease, and it is naive to assume all patients’ tumors share the same metabolic addictions. The prognostic gene signatures related to lipid metabolism, polyamines, or hypoxia discussed previously are a crucial first step, as they confirm the clinical relevance of these pathways. A polyamine-associated gene panel (e.g., FAM162A, SIGMAR1, PYCR1) identifies immune-suppressed phenotypes and supports DFMO-based regimens (107). Likewise, hypoxia- or lactate-metabolism–related gene signatures in OS predict prognosis and mirror immune contexture, offering a basis to infer potential responsiveness to immunotherapy (154). However, the field must move from prognostic models to predictive biomarkers. Recent advances in programmable endonuclease–assisted ctDNA assays enable ultrasensitive detection of rare variants (≤0.1% mutant-allele frequency), providing a template for minimal-residual-disease tracking and OS-relevant alterations (155). Consistent with this direction, ferroptosis-based mRNA/lncRNA signatures in human cancers show prognostic utility and correlate with immune contexture, underscoring the translational potential of immunometabolic biomarkers for patient stratification (156). A successful clinical path forward will require:

- Patient Stratification: Designing clinical trials that enroll patients based on the specific metabolic phenotype of their tumor. For example, a trial for a GLS1 inhibitor should enroll patients whose tumors show high glutamine dependency via PET imaging or specific gene expression signatures.
- Biomarker-Driven Trial Design: Future studies should adopt designs, such as basket trials, where patients with different cancer types but a shared metabolic vulnerability (e.g., PHGDH amplification) are treated with a targeted inhibitor. This approach could accelerate the identification of responsive patient populations in a rare cancer like OS.

In conclusion, while the therapeutic arsenal targeting OS metabolism is expanding, progress requires a strategic shift towards rationally designed combination therapies, enabled by advanced delivery technologies, and guided by robust, predictive biomarkers.

## 5 Conclusions and perspectives

The study of OS is at a pivotal juncture. Emerging evidence compels us to reframe this malignancy not merely as a disease of uncontrolled proliferation, but as one fundamentally orchestrated by a deeply intertwined network of metabolic reprogramming and immune evasion. The dysregulation of glucose, lipid, and amino acid pathways, amplified by the pervasive hypoxia of the bone microenvironment, converges to create a uniquely hostile landscape for antitumor immunity. While preclinical research has successfully identified a multitude of actionable metabolic vulnerabilities, a critical and honest assessment of the field reveals a significant gap between this preclinical promise and the current clinical reality.

### 5.1 A critical assessment: the metabolic underpinnings of immunotherapy resistance in osteosarcoma

A central paradox in osteosarcoma treatment is the profound disconnect between its high mutational burden—a feature typically associated with responsiveness to immunotherapy—and the deeply disappointing efficacy of immune checkpoint inhibitors in clinical practice (157, 158). Why has immunotherapy, a revolutionary treatment for many other cancers, largely failed in OS? This review posits that the answer lies not in the absence of antigens, but in the tumor’s mastery of metabolic reprogramming to erect a series of formidable barriers against the immune system. This metabolic defense strategy explains the failure of immunotherapy on several levels:

Constructing a “Cold” and Metabolically Hostile TME: OS is often described as an immunologically “cold” tumor, characterized by a paucity of T-cell infiltration. This is not a passive state but an actively maintained one. As detailed in this review, the relentless glycolysis of OS cells leads to glucose deserts and lactate seas—a toxic microenvironment that starves and exhausts infiltrating T cells long before they can mount an effective attack. Furthermore, a dysregulated lipid environment can directly trigger T-cell ferroptosis. Therefore, even if checkpoint inhibitors “release the brakes” on T cells, these cells are metabolically crippled and lack the fuel or functional integrity to respond.

Extreme Metabolic Heterogeneity and Plasticity: OS is a disease of extreme genomic and, consequently, metabolic heterogeneity (159). Different tumor subclones may rely on different metabolic addictions (e.g., some are glycolytic, others rely on FAO). Furthermore, cancer cell plasticity—the ability of tumor cells to dynamically switch phenotypes—is a key driver of this heterogeneity and therapeutic resistance. This phenotypic switching allows subpopulations to survive targeted therapies by adopting transient, drug-tolerant states, which further complicates treatment (160).This creates a mosaic of metabolic challenges for the immune system and means that there is no single, uniform immunosuppressive mechanism to target. This explains the inconsistent and often poor responses to therapies aimed at a single pathway.

The “Extrapolation Gap” as a Barrier to Progress: Our ability to solve the problem of immunotherapy resistance is hampered by an over-reliance on preclinical models that do not faithfully recapitulate OS immunometabolism. As we have critically highlighted, many immunomodulatory mechanisms are assumed based on work in carcinomas. The lack of robust, immune-competent OS models has led to a critical “extrapolation gap,” slowing the discovery of OS-specific metabolic vulnerabilities that could be targeted to truly unleash the immune system.

### 5.2 A roadmap for the future: towards precision immunometabolic therapy

Aligning with the broader shift toward personalized and precision medicine in oncology (161), overcoming these challenges requires a strategic, multi-pronged approach that moves beyond simplistic, single-target interventions. We propose the following roadmap to guide future research and accelerate clinical translation:

Mapping the OS Immunometabolic Atlas: The immediate priority is to systematically map the metabolic landscape of OS using multi-omic technologies. Integrating spatial transcriptomics, proteomics, and metabolomics on patient samples will allow us to define distinct immunometabolic subtypes, identify novel therapeutic targets, and uncover mechanisms of resistance. This will finally move the field from extrapolation to OS-specific discovery.

Developing Better Models: There is an urgent need for more sophisticated preclinical models, such as syngeneic or genetically engineered mouse models (GEMMs) of OS that possess a fully competent immune system, or advanced patient-derived organoid (PDO) co-culture systems that incorporate immune cells. These models are indispensable for validating targets and testing combination therapies in a more relevant context.

Designing Smarter Clinical Trials: The era of empirically testing metabolic inhibitors in unselected patient populations must end. Future clinical trials must be biomarker-driven. This involves using advanced imaging (e.g., hyperpolarized ¹³C MRI) and molecular profiling to stratify patients and enroll only those whose tumors exhibit the specific metabolic vulnerability being targeted (162). Adaptive trial designs, which allow for modification based on real-time metabolic monitoring of response, should also be explored.

In conclusion, immunotherapy resistance in OS is not merely a failure of antigen recognition but reflects a deeply embedded metabolic defense system. Overcoming this requires abandoning extrapolated assumptions and investing in OS-specific discovery frameworks. By integrating multi-omic profiling, advanced modeling, and precision trial design, the field can transition from mechanistic insight to clinically actionable immunometabolic interventions, ultimately improving outcomes for OS patients.
